# Molecular and Circulating Biomarkers in Patients with Glioblastoma

**DOI:** 10.3390/ijms23137474

**Published:** 2022-07-05

**Authors:** Nadia Senhaji, Asmae Squalli Houssaini, Salma Lamrabet, Sara Louati, Sanae Bennis

**Affiliations:** 1 Department of Biology, Faculty of Sciences, Moulay Ismail University, Meknes 50000, Morocco; 2 Laboratory of Biomedical and Translational Research, Faculty of Medicine, Pharmacy and Dental Medicine of Fez, Sidi Mohamed Ben Abdellah University, Fez 30070, Morocco; asmae-squalli_houssaini@hotmail.com (A.S.H.); salma.lamrabet@usmba.ac.ma (S.L.); sabennis@yahoo.fr (S.B.); 3 Medical Biotechnology Laboratory, Faculty of Medicine and Pharmacy of Rabat, Mohammed Vth University, Rabat 10000, Morocco; sara.louati@yahoo.fr

**Keywords:** glioblastoma, molecular biomarkers, liquid biopsy

## Abstract

Glioblastoma is the most aggressive malignant tumor of the central nervous system with a low survival rate. The difficulty of obtaining this tumor material represents a major limitation, making the real-time monitoring of tumor progression difficult, especially in the events of recurrence or resistance to treatment. The identification of characteristic biomarkers is indispensable for an accurate diagnosis, the rigorous follow-up of patients, and the development of new personalized treatments. Liquid biopsy, as a minimally invasive procedure, holds promise in this regard. The purpose of this paper is to summarize the current literature regarding the identification of molecular and circulating glioblastoma biomarkers and the importance of their integration as a valuable tool to improve patient care.

## 1. Introduction

Gliomas constitute the most common type of tumors of the central nervous system. They represent approximately 30% of all primary brain tumors and 80% of all malignant tumors [1]. They are initially classified, based on their cell of origin, into astrocytoma, oligodendroglioma, or ependymoma. Then, the establishment of the degree of malignancy according to the World Health Organization (WHO) classification criteria allows the organization of these tumors into grades ranging from I to IV. In 2016, the WHO classification of tumors of the central nervous system introduced molecular biology, for the first time, as an essential tool for the characterization of many molecular markers of diagnostic and/or prognostic interest [2]. The fifth edition of the WHO classification criteria (2021 version) focuses more on the involvement of molecular biomarkers in tumor classification [3].

According to the CBTRUS Statistical Report, glioblastoma is the most commonly occurring malignant central nervous system tumor (14.5% of all tumors). Despite the considerable advances in the diagnosis and treatment of tumors in recent decades, GBM remains one of the deadliest human cancers. Indeed, GBM presents the lowest median observed survival of all primary malignant brain and other CNS tumors (8 months) [1]. This poor prognosis is mainly caused by therapeutic resistance and recurrence after surgical removal. Current therapeutic approaches for GBM combine surgery, radiotherapy, and chemotherapy. Still, even with the use of Temozolomide, the standard chemotherapeutic drug, GBM patients show a low median survival of ~15 months [4]. Some immunotherapies are being tested in several clinical trials and might improve GBM treatment [5,6].

Nowadays, the diagnosis of glioblastoma is mainly based on neuroimaging and tumor resection [4]. Biological material can be obtained either from a tissue biopsy or a liquid one. While tissue biopsy remains the standard procedure commonly used for the histological characterization of glioblastoma, liquid biopsy has recently emerged as a promising way to improve patient care in terms of initial diagnosis, relapse, and choice of appropriate treatments. In fact, liquid biopsy provides the advantage of being a minimally invasive procedure that overcomes the high risks of surgery or even, in some cases, the impossibility of performing it [7]. Furthermore, the most important hallmark of GBM is tumor heterogeneity [8]. Hence, the need to rely on new approaches that might describe the evolution of the tumor and avoid a static characterization of an ever-changing tumor is primordial.

The aim of this review is to provide an overview of the molecular and circulating biomarkers necessary for the diagnosis and monitoring of GBM. This paper also explores the new advances in GBM therapy.

## 2. Molecular Biomarkers

### 2.1. IDH

*IDH* mutations play a crucial role in glioma classification and have great prognostic significance [9,10]. Patients with the *IDH* mutant present a better probability of survival than patients with *IDH* wild-type glioma due to a younger average age at diagnosis [11,12,13]. Several studies have demonstrated that resection of the entire tumor correlates with a better prognosis in patients with mutated IDH1, compared with the wild type [14,15,16,17].

Isocitrate dehydrogenase (*IDH*) catalyzes the oxidative decarboxylation of isocitrate to alpha-ketoglutarate during the Krebs cycle and is the primary source of cytosolic NADPH production in the human brain and other tissues. *IDH1* (2q33) and *IDH2* (15q26) are also involved in protecting against replicative senescence by reducing lipid peroxidation in cell culture and oxidative DNA damage.

IDH mutation occurs in the early stage of gliomagenesis [18,19]. Glioblastomas with wild-type *IDH* are predominantly primary or de novo and arise early in patients over 50 years of age. These patients are characterized by a short clinical history, usually less than three months before diagnosis, and with no pre-existing lower-grade glioma [2].

In contrast, glioblastomas with mutated *IDH* are generally seen in young adults and include mostly secondary glioblastomas that can develop as a result of the evolution of a pre-existing grade II or III glioma. They represent approximately 10% of all glioblastomas [2,20]. Secondary glioblastomas are described by the arginine–histidine substitution at codon 132 of *IDH 1* (c.395 G > A) [2,20]. However, others express *IDH 2* mutations located at codon 172, and arginine is substituted by lysine (c.515 G > A).

*IDH1* and *IDH2* mutation occurrences are mutually exclusive [21]. These mutations reduce alpha-ketoglutarate, the normal product, to D-2-hydroxyglutarate (D2HG) to consume NAPDH and participate in gliomagenesis. D2HG may be an oncometabolite that competitively inhibits alpha-ketoglutarate-dependent enzymes, which play an important role in gene regulation and tissue homeostasis [22].

However, the process by which D-2HG induces tumorigenesis is still unknown, but it is probably related to the effects of D-2HG on DNA demethylases, causing DNA and histone methylation. In addition, alpha-ketoglutarate levels influence the hypoxia-inducible transcription factor HIF-1α, which promotes tumor growth when oxygen levels are low.

In addition, the level of 2-HG was assessed using magnetic resonance spectroscopy and found to be a relevant biomarker for monitoring treatment response [23,24]. A molecular study of *IDH1* (exon 4) and *IDH2* (exon 4) by DNA sequencing is required to exclude the presence of other *IDH1* or *IDH2* mutations [2].

The *IDH* mutation can be detected by immunohistochemistry using an antibody directed against the *IDH1* R132H mutation [19,25]. This method detects the missense mutation and is very reliable in different laboratories [26]. The sensitivity and specificity of the immunohistochemistry technique with the anti-IDH R132H antibody can reach 100% [25].

Given the impact of the *IDH* mutation, the inhibition of mutant IDH1/2 is a promising treatment for gliomas. Several clinical trials are underway [27,28]. Nevertheless, the effects of *IDH* inhibitors are controversial. Some studies have reported that *IDH* inhibitors inhibit the proliferation of *IDH1* mutant glioma cells [29] while other studies find it ineffective [30].

### 2.2. MGMT

The MGMT (O6-methylguanine DNA methyltransferase) gene is located at 10q26. It codes for a DNA damage repair protein that plays a major role in the repair of alkyl adducts at the O6 position of guanines [31,32,33].

The methylation of the MGMT promoter leads to a loss of expression of the MGMT by reducing DNA repair activity. As a result, the tumor is more chemosensitive to alkylating agents such as temozolomide [31,32,33].

This methylation induces an inaccurate pairing of the methylated guanine with the thymine that has just been incorporated during replication. The mismatch repair (MMR) system, responsible for removing the thymine but leaving the methylated guanine, causes double-strand breaks in the DNA, irreparable damage to the genome, and activates cell death [34,35,36].

To contain this action, MGMT removes and transfers the methyl group from the O6-MeG position to a cysteine residue irreversibly. This effectively reverses alkylation-induced DNA damage and attenuates the cytotoxic effects of temozolomide. This mechanism explains why patients with MGMT-expressing cancer cells generally do not respond to temozolomide therapy [37,38].

To observe the MGMT methylation status in GBM patients, nested polymerase chain reaction (PCR) or combinatorial PCR with MS (methylation-specific PCR) [39], SYBR Green [40], or even pyrosequencing are used [41].

MGMT promoter methylation is found more often in secondary glioblastomas than in primary glioblastomas (75% versus 36%) [42,43].

Several studies have shown that MGMT methylation corresponds to better overall survival and progression-free survival (probably with *IDH* mutations) in patients treated with alkylating agents. In glioblastoma, the combination of *IDH* and MGMT status is more predictive of survival than *IDH* or MGMT alone [44]. These mutations are associated with better response rates to TMZ [17,45].

In addition, Roszkowski and his team found that patients with *IDH* mutations and MGMT promoter methylation receiving radiation therapy had a better prognosis than those with MGMT methylation alone [46].

The survival time and progression-free survival of glioblastoma patients with *IDH* mutations alone was shorter than that of patients with *IDH* mutations and MGMT methylation [17,44,47,48].

Nevertheless, another study reports that only patients with TERT promoter mutation combined with MGMT methylation may benefit from temozolomide and be sensitive to its activity [49]. Additionally, Nguyen and al. report that patients with MGMT methylation had improved survival only in the presence of TERT promoter mutations [50].

The dual inactivation of MGMT by the loss of 10 q and promoter methylation induces a greater sensitivity to TMZ treatment than the absence of 10q or promoter methylation alone [32].

All these studies show that MGMT methylation represents a good prognostic marker in glioblastoma. It is very useful to establish different approaches in glioblastoma patients based on their MGMT status, and to introduce MGMT biomarker assessment into routine clinical practice.

### 2.3. Epidermal Growth Factor Receptor

EGFR is a transmembrane receptor with tyrosine kinase activity involved in several signaling pathways such as the (PI3K/AKT/mTOR) pathway that promotes proliferation, cell survival through the progression of the cell cycle and the inhibition of apoptosis, and the (Ras/Raf/MAPK) pathway that drives cell differentiation, proliferation and migration [51,52]. These signaling pathways inhibit apoptosis and reduce the efficacy of temozolomide treatment [51,53]

The most common genetic alterations present in approximately 57% of glioblastomas are mutations, alternative splicing, rearrangements, and the focal ample of EGFR [54].

EGFR is amplified in approximately 60% of glioblastomas. This amplification is found almost exclusively in patients with primary glioblastoma, and is very rare in secondary glioblastomas (Wang et al., 2015).

Several studies have shown that *IDH* mutations and EGFR amplification are mutually exclusive [55,56]. They are often accompanied by overexpression; 97.7% of glioblastomas with non-amplified EGFR do not show EGFR overexpression [57,58]. The mutation of EGFR due to histone modifications on the enhancer of its gene located at chromosome 7p12 and exons 2 and 7 leads to the formation of EGFRvIII [58].

This rearrangement is the most common variation of EGFR and is found in almost 50% of these cases, causing an increase in its activity. In fortification, EGFRvIII is characterized by a 267 amino acid deletion in the extracellular domain, which results in a lack of binding, constitutive activation, and the proliferation of glioblastomas via protein kinase (PKA)-dependent activity [57,59,60].

The alteration of EGFR can be detected by fluorescence in situ hybridization (FISH), immunohistochemistry, and real-time PCR (qPCR) [55,61].

Studies have shown that EGFR amplification alone has no prognostic impact on survival [42,62]. However, Shinojima N et al. have noted a significantly shorter survival in patients with EGFR overexpression. Moreover, the prognosis is even worse when this alteration is accompanied by amplification [58]. Patients without an EGFRvIII mutation have significantly longer survival (1.4 years) than patients with a mutation (0.8 years) [58].

Montano et al. noted a significantly longer overall survival in GBM patients with EGFRvIII treated with total resection and standard radiochemotherapy (Temozolomide). In addition, EGFRvIII/Ki67 (20% or less) and EGFRvIII/methylated MGMT combinations positively impacted the prognosis of glioblastoma patients [63].

Another study showed that, in combination with the TERT mutation, patients with wild-type EGFR had an average survival twice that of patients with EGFR amplification [64]. Furthermore, glioblastoma patients with wild-type EGFR had longer survival with wild-type TERT than patients with wild-type EGFR and mutated TERT [65].

Up to now, various drugs have been developed to target EGFR. The latter’s monoclonal antibodies such as cetuximab and nimotuzumab have been studied as anti-tumor agents, as well as tyrosine kinase inhibitors (TKIs) targeting signal transduction, such as afatinib, gefitinib, and erlotinib. Unfortunately, no inhibitors have been approved for glioblastoma [66,67,68]. This is probably due to the presence of the blood–brain barrier that prevents access to resistance-promoting mutations [69].

EGFRvIII has also been targeted by vaccination approaches, using a unique antigenic epitope from the mutant protein sequence. The rindopepimut vaccine trial for newly diagnosed glioblastoma did not show a survival benefit in phase 3 [15,70].

### 2.4. TERT

Telomerase is a ribonucleoprotein enzyme complex that maintains and extends telomeres in eukaryotes using a native RNA molecule as a template. Therefore, it can extend the number of cell divisions and act as an RNA-dependent DNA polymerase that compensates for the loss of these DNA sequences by producing telomeric replicas in cells capable of division [71].

Somatic mutations in TERT involving regulatory regions, in addition to coding sequences, may be involved in oncogenesis [72]. In addition, mutations in the TERT promoter reactivate telomerase, which prevents telomere shortening and leads to the immortalization of tumor cells [73].

This alteration is a biomarker that can provide additional diagnostic information. According to the fifth edition of the WHO Classification of Tumors of the Central Nervous System (WHO CNS5), the detection of this alteration allows the reclassification of an *IDH* wild-type astrocytoma of grade 2 or 3 into an *IDH* wild-type glioblastoma [10].

Approximately 70–90% of glioblastomas have mutations in the TERT promoter (C228T or C250T) located at −124 bp and −146 bp upstream of the TERT translation start site (5p15.33) [74]. The mutations were cytosine to thymine transitions: 1295228 C > T and 1295250 C > T) [74]. The C228T mutation accounts for 75% and the C250T for 25% of all TERT promoter mutations [75].

These alterations can be detected by MS PCR, Sanger, mass spectrometry-based assays, next-generation sequencing (NGS), and digital droplet PCR (ddPCR) [76].

Several studies have shown that the prognostic significance of TERT mutations depends on age, tumor histology, surgery, *IDH* wild status, and unmethylated MGMT promoter status [50,74,77,78]. The TERTp mutation is a favorable prognostic factor in *IDH*-mutant glioblastoma. [64,65]. In addition, the patients with TERTp and unmethylated MGMTp mutations have the worst prognosis. TERT promoter mutation is a poor prognostic factor in *IDH* wild-type glioblastomas [74,79,80].

Given the high frequency of TERT mutations in glioblastoma, strategies inhibiting telomerase activity may present an attractive therapeutic target, namely inhibitors, immunotherapy, and vaccines. Despite many promising results, no therapy has been able to demonstrate clinical efficacy in the management of patients with glioblastoma [72].

### 2.5. LOH: Loss of Heterozygosity

According to CN5S (2021), the diagnosis of *IDH* wild-type glioblastoma is based on the presence of EGFR amplification or TERT promoter mutation, or the combined gain of the whole chromosome 7 and the loss of whole chromosome 10 (+7/−10) [3,81,82,83].

LOH primarily affects tumor suppressor genes such as PTEN and *TP53*, which results in the decreased protection of the body′s systems against tumorigenesis [84,85].

Glioblastomas often have a loss of heterozygosity, particularly on chromosomes 7, 9p, 10, 17p, 19q, and 22 [85,86,87]. The gain of whole chromosome 7 is associated with a 4.7 fold increased risk of tumor recurrence [88].

LOH 10q23 is present in 70–80% of primary glioblastomas [3,81,82,83] and LOH 10q25qter is useful for the diagnosis of secondary glioblastomas [89].

Thus, LOH 10 alteration is involved in the pathogenesis of both primary and secondary glioblastomas. Moreover, LOH 10 is present in 84.2% of patients aged 40 years and older versus 16.7% of patients aged 40 years and younger [89].

The loss of chromosome 10q is a form of MGMT inactivation located on chromosome 10q26. One study showed that glioblastoma patients with the dual inactivation of MGMT, by loss of the long arm of chromosome 10 and the hypermethylation of the MGMT promoter, have longer progression-free survival and overall survival, and respond well to temozolomide therapy [32,79].

LOH 10 has also been observed in 40% to 80% of glioblastomas with p53 mutation and 60% to 100% of glioblastomas with EGFR amplification [90]. Hata and his team believe that LOH 10 has a very important prognostic biomarker for primary and secondary GBM [89]. Studies have shown that patients with no alterations in chromosome 10 have longer survival than those with LOH 10 [91]. LOH 19q is frequently involved in the progression of low-grade astrocytoma to secondary GBMs [92].

LOH 1p has been detected in 10% of GBMs [91,92,93], while LOH 13q has been recorded in 35% of GBMs [85,94,95]. LOH 22q is an alteration present in 11–39% of gliomas, but more frequent in glioblastomas. This strongly suggests a link between this alteration and tumor progression [33].

Gains in chromosome 7 and alterations in chromosomes 9 and 17 are also common in glioblastomas. They are associated with the amplification of oncogenes such as EGFR, as well as the mutation and/or deletion of tumor suppressor genes such as *TP53* [52,96].

### 2.6. *TP53*

The *TP53* gene is located on chromosome 17q13.1 and encodes p53. The *TP53*/*MDM2*/*MDM4*/*p14AR* pathway is involved in differentiation, apoptosis, cell cycle regulation, DNA damage response, and genomic stability [97,98].

P53 is a well-known transcription factor and antioncogene and is involved in most cancers, including glioblastoma [97]. *TP53* can be inactivated indirectly after deletion or mutation, or directly due to the alteration of its associated genes. Based on TCGA data, 78% of glioblastomas have mutations in the *TP53*/*MDM2*/*MDM4*/*p14AR* pathway. These alterations often coexist with the *IDH* mutation and may promote progression from low-grade astrocytoma to glioblastoma [98].

Therefore, 35% of patients are affected by mutations or the homozygous deletion of *TP53*, 14–20% due to amplification of *MDM2*/*MDM4*, and 49% following homozygous deletion or the mutation of *p14AR*F (Cancer Genome Atlas Research Network, 2008).

The accumulation of the mutated protein p53 is the core of tumor cells [99]. This has been observed in 59% of low-grade astrocytomas and 53% of anaplastic astrocytomas [100]. It also characterizes 30% of primary glioblastomas (mostly at exons 5, 6, 7, and 8) and 65–90% of secondary glioblastomas at codons 248 and 273 (Exons 7 and 8) and in CpG sites (methylation region) [98,100,101].

The presence of these mutations at different grades suggests that they occur early in gliomagenesis and accumulate as the glioma progresses [100,102]. The p53 mutations seen in primary glioblastomas may occur as a secondary event resulting from genomic instability in the glioblastoma tumor microenvironment [52,100]. These mutations can be detected by immunohistochemistry and the mutation of the *TP53* gene can be detected by qRT-PCR.

MGMT promoter methylation is highly correlated with *TP53* mutation (92%) [102]. Indeed, p53 can downregulate MGMT via an interaction with the transcription factor Sp1 [103]. Clinically, glioblastoma patients with lower MGMT expression have a better prognosis with p53-expressing phenotypes [104]. In general, *TP53* mutation is associated with a bad prognosis, but it is still largely undetermined in glioblastomas. P53 represents a viable therapeutic target for the treatment of glioblastoma [98].

In an attempt to overcome the p53 mutation, wild-type p53 expression can be increased in tumor cells using adenovirus-mediated p53 gene therapy that can appropriately arrest cell progression at checkpoints. As a result, cell proliferation, tumorigenesis, and progression in vivo will significantly decrease [98].

In a phase I trial, Lang and colleagues were able to show that the intra-tumoral injection of an adenoviral vector containing p53 resulted in *TP53* gene transfer and the expression of functional exogenous p53 in all patients. The transfected cells were found near the injection site, indicating the absence of systemic viral dissemination [105].

Other studies have attempted to evaluate the efficacy of transduction and the effectiveness of wild-type *Ad5CMV-p53* gene therapy (trial NCT00004041) or recombinant adenovirus-p53 SCH-58500 (NCT00004080) in combination with surgery. However, there is potential resistance and poor gene transfer [98].

Several studies using drugs that attempted to facilitate the folding of the mutant proteins into their wild-type conformation were not effective. Other trials targeting *MDM2* and *MDM4* neutralization for glioblastoma patients with impaired p53 function after *MDM2* or *MDM4* gene amplification are underway (NCT03107780) [80].

### 2.7. *ATRX*

The *ATRX* gene is linked to *ATRX* syndrome (i.e., X-linked mental retardation syndrome). The *ATRX* protein exists in two isoforms (180 and 280 KDa) and is highly enriched in GC-rich palindromic sequences. The C-terminal end of the *ATRX* protein contains the helicase/ATPase domain classifying *ATRX* in the SNF2 family of chromatin remodeling proteins. Besides the N-terminal domain presents the ATRX-DNMT3-DNMT3L (ADD) with cysteine-rich motifs and similar characteristics to those of DNMT3 proteins involved in methylation. Furthermore, *ATRX* is a regulator of chromatin remodeling and transcription by establishing an ATP-dependent complex with a *DAXX* transcription cofactor. In addition, *ATRX* also modifies the DNase I digestion pattern and triple-helix displacement activity and participates in cell cycle regulation and the maintenance of genomic stability [106].

*ATRX* mutations are detected through immunohistochemistry staining (the lack of nuclear staining in this case) which are relatively associated with an ATL (i.e., alternative lengthening of telomeres) phenotype. The loss of *ATRX* prevents the recruitment of the protein *DAXX* in the telomeric region of the chromosome. The complex ATRX-DAXX-H3.3 is inhibited by the *ATRX* alteration, affecting the process of DNA remodeling. As a consequence, the compaction of the chromatin will be reduced and reachable for the transcription enzymes that can lead to the transcription of different genes including oncogenes. TEERA is an enzyme activated in this process creating an R-loop that escorts the telomere lengthening process alongside TERT triggering, which is highly implicated in glioblastomas [107].

*ATRX* mutation is mutually exclusive with the 1p/19q co-deletion; thus, this mutation is characteristic of secondary glioblastoma from an astrocytic descent [9]. The correlation established between *IDH* and *ATRX* in GBM expresses that there is a loss of function in *ATRX* in 75% of *IDH*-mutant secondary GBM. However, the rate of patients expressing *IDH* wild type associated with *ATRX* loss is 3%, pronouncing a better overall survival compared to *IDH*-mutated and *ATRX*-WT [107,108].

### 2.8. VEGF

Vascular endothelial growth factor VEGF (VEGF-A) is a member of a family of proteins including VEGF-B, VEGF-C, VEGF-D, and VEGF-E [109]. VEGF-A has been detected as a primary mediator of angiogenesis and tumor progression through the activation of phosphatidylinositide-3 kinase (PI3K)/Akt-dependent or RAS/ERK signaling pathways [110].

VEGF’s overexpression has been detected by immunohistochemistry in 64.1% of glioblastomas, triggering efforts to develop the anti-VEGF drug “Bevacizumab” [111,112,113]. This is an anti-angiogenic therapy that can block tumor vascularization by neutralizing VEGF-A overexpression [114].

This antibody was approved by the FDA and used as a treatment of glioblastoma based on encouraging progression-free survival results and high radiological response rates in pre-clinical and clinical (phase II) trials [111,115,116,117]. However, bevacizumab did not improve overall survival in patients with glioblastoma [115,117,118].

This failure may be explained by the complexity of pro-angiogenic signaling [119], and the activation of VEGFR by another ligand during bevacizumab treatment [120,121,122]. Moreover, the VEGF ligand family includes several ligands such as VEGF-C, which is overexpressed in glioblastoma and associated with tumor progression [64,123,124]. High levels of VEGF-C are a poor prognostic factor [125,126].

### 2.9. Ki-67

Ki-67 is an index of cell proliferation that ranges from 15% to 40% in most glioblastomas. It is mostly detected in high mitotic activity areas [127].

In neuro-oncology, the monoclonal antibody Ki-67 is widely used [128,129,130]. It reacts with nuclear proteins expressed in the GI, S, G2, and M phases of the cell cycle. Nuclear positivity for Ki-67 is determined by using IHC, counting at least 1000 tumor cells in a homogeneously stained area. Cases with <10 and ≥10% stained cells are defined as negative and positive, respectively [131].

Rashmi et al. enrolled 83 patients with GBM presenting Ki67 positivity; they also reported a high expression of Ki-67 in *IDH* wild-type gliomas [132]. Tokano et al. showed that a glioma with wild-type *IDH* and Ki-67 ≥10% strongly suggests the diagnosis of glioblastoma. A high expression of Ki-67 is associated with lesion volume, increased risk of recurrence, and consequently worse prognosis [131].

Other studies have reported that the presence of low ki-67, methylated MGMTp, unmutated TERTp, and mutated *IDH* in young patients have a positive synergistic effect on survival. These results may suggest that glioblastoma survival depends on a combination of intrinsic patient characteristics and genetic mutations [78,133,134].

Using Ki-67 reflects the aggressiveness of tumor phenotypes. Currently, the use of Ki67 varies between the prognostic stratification of patients and resistance or sensitivity to chemotherapy [135,136,137].

Similarly, Bredel et al. proposed that tumors with increased proliferation are more prone to the cytotoxic effects of chemoradiotherapy [138]. Thus, therapeutic decisions should be guided by clinically relevant prognostic factors such as Ki-67 expression, which could be a predictive factor of poor prognosis in gliomas [139].

Some studies suggest a relationship between a higher level of the Ki67 index and a longer life span [128,129,130,138], while others demonstrate that this parameter has no value in determining the prognosis of glioblastoma [135,140]. To overcome this discrepancy, it is necessary to standardize the methods of quantification of this index, which suffer from inter-and intra-observer variability [135,141,142,143].

### 2.10. MMR

Several research projects have focused on understanding the involvement of DNA repair mechanisms in the DNA damage response (DDR), which has been investigated in multiple solid tumors such as glioblastoma. Mismatch repair is an extremely conserved damage repair mechanism due to its fundamental approach in preserving genome integrity. The MMR process has been described as the pathway targeting base substitution, as well as other mismatches developed due to DNA replication errors [144]. The loss of this mechanism has been associated with microsatellite instability (MSI) (i.e., a genomic condition of hypermutability with a phenotype related to the loss of MMR) [145].

This pathway has been investigated through its eight proteins (i.e., MSH2, MSH3, MSH5, MSH6, MLH1, PMS1, MLH3, and PMS2). The eight proteins operate in heterodimers (MSH2–MSH3) that recognize a single base mismatch and (MLH1- PMS2) lead the degradation and resynthesis (Leelatian et al., 2021). Mismatch repair loss is investigated alongside MGMT promoter silencing. O^6^-methylguanine expression is a result of a cytotoxic disturbance directed by the alkylation effect of prior GBM treatment. The mutational load is established by a defective MMR pathway [6]. Through The Cancer Genome Atlas (TCGA) program, it has been identified that the mismatch occurring in the CpG islands located in MGMT promotor is a substitution of C *G by A *T. This mutation will be settled due to the MMR pathway defect [6]. Notably, MSH6 inactivation associated with microsatellite instability displays a tolerant aspect of tumor cell growth induced by therapy [144].

Proteins of the MMR pathway have been explored by immunohistochemical staining by several antibodies. Other techniques can be used such as next-generating sequencing for molecular detecting and fluorescent PCR and capillary electrophoresis in favor of detecting MSI [146].

The majority of GBM diagnosed present a high mutational burden associated with MMR deficiencies, MSI development, or mutations in the *POLE* gene (i.e., encoding the catalytic subunit of DNA polymerase epsilon, an enzyme that plays a critical role in DNA replication and repair). However, defective MMR has been correlated to sporadic alterations, particularly, *BRAF* V600E mutations and/or *MLH1* promoter silencing or germline alterations, such as *MLH1* gene mutations [147].

Considering the impact of alkylators on the installation of a defective mismatch repair mechanism and microsatellite instability, some researchers have described potential immunotherapy for patients with biallelic MMR mutation using an anti-PD1 antibody [148]. Patients without prior alkylator treatment demonstrating germline mutations have displayed immunotherapy efficacity [149]. Accordingly, cases with recurrent GBM had expressed a limited efficacity with an OS of 14.4 months [144].

### 2.11. PD-1 and PDL-1

Divergent immune checkpoints are a principal factor abused by tumors to escape the targeting by the immune system [150]. The programmed cell death protein 1 (PD-1) encoded by the *PDCD1* gene is a transmembrane receptor expressed in different types of cells including microglia, antigen-presenting cells (APC), and B lymphocytes. It is activated by its ligand PD-L1 (i.e., programmed cell death protein ligand 1), a protein encoded by the *PDCDL1* gene. All along this activation process, several proteins are entangled by phosphorylation and dephosphorylation mechanisms in which tyrosine phosphatase protein is triggered to lead to Zap70 dephosphorylation [151]. As a result, a downregulation of the T lymphocytes and their cytotoxicity associated with the production of cytokines such as IFN-γ is a predominant factor in the pro-inflammatory process [150].

In all gliomas, GBM expresses a higher rate of PD-1- and PD-L1-associated forms to tumor-infiltrated lymphocytes (TIL) compared to other grades. This tumorigenesis process is related to the capacity of TIL to present PD-1 on their surface, impersonating normal $L_{T}$. Hence, they become unrecognizable by the immune system. In addition to its mechanisms, the couple PD-1/PD-L1 is associated with fundamental cell signaling pathways such as EGFR, IDH, and VEGFR. The correlation established between EGFR and PD-1 in glioblastoma is affiliated to the expanded rate of EGFR amplification due to the expression of PD-1. PTEN is a relevant protein in this signaling pathway, being the expression regulator of PD-L1. Consequently, the downregulation of the PI3K/AKT/mTOR signaling in the GBM microenvironment by the expression of PD-1 leads to an immune-resistant phenotype [151]. *IDH* is another relevant biomarker investigated in GBM tumorigenesis. Its manifestation is correlated to the expression of PD-1 and TIL expansion. The *IDH* wild-type tumor cells present an activated form of the immune system and a higher level of PD-1 expression [150].

VEGF is a well-known marker implicated in the inhibition of the maturation of dendritic cells promoting a defective antigen presentation translating immunosuppressive features [150]. Several studies have shown that the couple PD-1/PD-L1 implies the angiogenic mechanism alongside VEGF; it shifts from immunosuppressive to immune-supportive in the tumor microenvironment. According to Xue and et al., the expression levels of PD-1 correlate with VEGF. Nevertheless, Joseph along with his team present in their data a negative interconnection between the two units [152,153]. This contradiction provides a new arena for further investigations considering the significance given to these relevant markers.

These biomarkers have been identified by immunohistochemical staining in several studies [150,153].

The couple PD-1/PD-L1is implicated in the bio-stratification of GBM, and one of the mainly discussed agents for therapeutic purposes. Immunotherapy is based on targeting several immune pathways to restore the lost T-cells activity due to the confusion driven by tumor cells. Nivolumab is the main monoclonal antibody used to block receptors and ligands in effector cells belonging to the immune system [154]. Nevertheless, the complicated combination of glioblastoma and its challenging character has been identified by Simonelli et al. as the “immune-deserted phenotype”; due to the immunosuppressive environment unaffected by single targeted therapy, a combined strategy is the greatest exit.

### 2.12. BRAF-V600E

*BRAF* is the v-RAF murine viral oncogene homolog B1 protein from the RAF family related to a serine/threonine kinase signaling pathway [155]. It is a protein involved in the mitogen-activated protein kinase/extracellular mutation-regulated kinase pathway [156]. The most common mutation in cancer related to this gene is $BRAF^{V600E}$. It results through the substitution of thymine-to-adenine at position 1799, leading to the substitution of glutamic acid to valine in the 600 position. This phenotype is translated by hyperactivity that might cause cell arrest in the presence of flawless repair mechanisms. Therefore, with deficient ones, it can be considered as a pro-oncogenic factor expressed in 50% of cases of epithelioid glioblastomas [157].

MAPK (mitogen-activated protein kinase) is a crucial regulator of the *BRAF* signaling pathway. Thus, MAPK inhibitors have displayed their efficacy, safety, and anti-$BRAF^{V600E}$ ability in several studies [158]. BRAFV600E mutation has not presented any significant reaction in favor of radiotherapy or chemotherapy [157].

However, targeted therapies against BRAFV600E have been associated with a better overall survival, especially within pediatrics patients and young adults (17–35 years). Everolimus and sorafenib are two drugs in phases 1 and 2 clinical trials, respectively, for recurrent glioblastomas targeting BRAF-signaling-associated pathways [69].

### 2.13. ROS and Hypoxia

Cellular redox balance represents a physiological balance between ROS (reactive oxygen species) and reduced equivalent that should be maintained in the cell compartment [159]. The oxidative stress molecules are typically described by a state of unbound charged molecules with oxygen-developing reactive properties, such as loose superoxide radical (O_2_−) and hydroxyl, as well as non-radical spices including hydrogen peroxide [160].

ROS are produced by multiple organelles included in various signaling pathways such as cell respiration, and stimulate the transcription of several genes. Mitochondria, endoplasmic reticulum (ER), and peroxisomes are the most highly implicated organelles in the process of oxygen species production [160]. The deficient coupling of ROS in the mitochondria during cellular respiration and the activation of the Warburg effect (i.e., the elevated level of glucose consumption and privileged lactate production even in normoxia conditions) is majorly supported in the malignancy of glioblastoma (GBM) [161]. In addition, hypoxia is a predominant feature involved in the development of the GBM microenvironment, considering its implication in the stability of HIF-1α. The latter is the hypoxia-inducible factor that has been identified as a major operator of the Warburg effect and the trigger in the occurrence of some solid tumors, including glioblastoma [162].

In normoxia conditions, oxygen levels range from 3.1% to 8.7%. Therefore, HIF-1 is hydroxylated by Fe^2+^ and 2-oxoglutarate-dependent dioxygenases (PHDs) in proline and asparagine residues, and then ubiquitinated. However, PHDs cannot work through hypoxic conditions, preventing the signaling pathway and leading to HIF-1α degradation. Thus, this protein is functional when it is accumulated and binds to another isoform HIF-1β. The complex targets the transcription of several key factors (e.g., plasminogen activator mitogen 1, vascular endothelial growth factor, VEGF receptor, etc.) [163], as well as regulating some pro-angiogenic chemokines such as stromal cell-derived factor 1α and chemokine receptor 4, along with the recruitment of endothelial cells improving proliferation [164]. This contributes to the development of GBM alongside the VEGF receptor, given its large involvement in the angiogenic signaling pathway, and the master regulator of glioblastoma expansion and aggressivity [163], otherwise known as hypoxia-induced angiogenesis [164].

HIF-1α is a prime driver in the regulation of glycolytic enzymes; it promotes the expression of PDK1 (pyruvate dehydrogenase kinase 1) that inhibits the pyruvate dehydrogenase implicated in glycolysis [159]. The limited accessibility of pyruvate to the Krebs cycle declines the rate of the oxygen consumed by mitochondria. This effect is linked to radiotherapy, which has been involved in the accumulation of ROS in solid tumors by the water radiolysis mechanism [160].

The effect of hypoxia can be seen through a distinguished standpoint considering the association between some epigenetic modifications and the accumulation of HIF-1α. These changes, such as protein folding in the ER (endoplasmic reticulum), are an important site for ROS production by 25% [159]. HIF-1α levels are also related to *IDH1* mutated forms, preventing their degradation, which can lead to a worse prognosis [161]. In a recent study, Sfifou et al. (2021) provided some relevant results on a Moroccan population in which they revealed, through an immunohistochemistry study, that patients with a negative expression of HIF-1α had improved overall survival. The group with “negative HIF-1α and positive IDH1” illustrated a better outcome with 85% survival rate at 12 months. Nevertheless, the patients with “positive HIF-1α and negative IDH1” expressed the worst prognosis with 18% survival [162].

Several studies have shown that oxygen-generated species resulting from irradiations are the main cause of the accumulation of electrons and protons in different cells. DNA ionization, directly or through ionized water molecules, is involved in double-strand breaks (DSB) and single-strand breaks (SSB) that spark the most poisonous form of DNA damage. In consequence, the combination of radiotherapy and chemotherapy produces a high level of free radicals influenced by mitochondrial modification, and induces an up-regulation of catalase and SOD-2. As a result, it leads to the accumulation of GSH (glutathione disulfide) which inhibits some drug’s effects and is involved in some chemotherapeutic resistance mechanisms [160].

### 2.14. MET

The *MET* gene encodes a proto-oncogene located on chromosome 7q 21–31 known as the HGF receptor. It allies with multiple tumor cell functions such as proliferation, survival, and mobility, and it particularly contributes to migration and invasion mechanisms, with 2 to 3% expression in GBM. However, it has a low standard activity in normal cells. c-MET is a protein investigated through immunohistochemistry. Normally, c-MET is a tyrosine kinase receptor expressed in epithelial and endothelial cells, but it exists in low levels in brain tissue. HGF is the only known ligand for this receptor. The coupling of c-MET and HGF activates multiple key signaling pathways such as MAPK and PI3K, since they altogether belong to the RTK (receptor tyrosine kinase) family-activated proteins [6].

**Figure 1:**
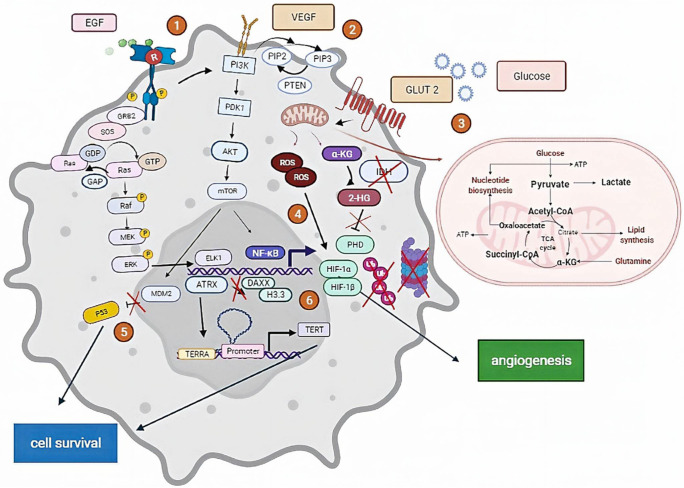
The main signaling pathways investigated in glioblastomas tumorigenesis.

The binding process between HGF and its receptor is affected by the autocrine or paracrine mechanisms. The aberrant c-MET form (i.e., a gain of expression, elevated paracrine and autocrine expression, mutation or gene amplification, and continuous receptor activation [165]) is correlated to primary and secondary GBM in 47% and 44%, respectively, of total confirmed cases. These results obtained by Q-PCR were reported by [166]. The autocrine signal is associated in some studies with a predictable sensitivity to HGF inhibitors. Furthermore, PTEN presents an affinity to HGF due to its main role as a co-regulator protein. This association reveals a certain response in therapy dictated by PTEN’s presence [167]. Several clinical trials have proceeded in MET inhibitor-based therapies. For instance, cabozantinib, a drug in phase 2 trials, is used for recurrent and progressive glioblastoma in children [69] (Figure 1).

(1) EGF receptors activate survival signaling pathways, including the RAS/MAPK and PI3K/AKT signaling pathways, leading to cell growth and proliferation. (2) The binding of VEGF to VEGFR-2 leads to form a receptor dimer and then activates the PI3K signaling pathway, including the activation of PTEN and the PKC-Raf kinase-MEK-mitogen-activated protein kinase (MAPK) pathway, which initiates DNA synthesis to promote endothelial cell proliferation. (3) The association between the glucose molecule and its receptor GLUT-2 activates mitochondrial mechanisms, including the production of ROS and α-KG; the latter produces 2-HG, inhibiting the ubiquitination of HIF-1α developed by ROS involved in angiogenesis (4). (5) P53 ubiquitination is related to *MDM2* activity. *MDM2* in mutated form inhibits P53 degradation, and the P53-mutated form is not recognized. (6) *ATRX* absence leads to the blockade of the complex ATRX-DAXX-H3.3, a lead transcription factor involved in the activation of TERT included in cell resistance and survival.

## 3. Circulating Biomarkers

### 3.1. Circulating Tumor Cells

The first discovery of tumor-free cells in blood was made by Australian researchers in 1869, more than a century ago. Circulating tumor cells (CTCs) are cancer cells released by the primary tumors into the bloodstream, disseminating at distant sites and eventually forming metastases. CTCs can exist in the form of single cells or clusters. The phenotypes of CTCs change after epithelial-to-mesenchymal transition, which leads to the loss of cell-to-cell contact and the development of less differentiated mesenchymal phenotypes or stem cells [168].

The concentration of CTCs in the blood circulation is very limited, with only 1 CTC per 10^9^ blood cells, which explains why their detection with high specificity and sensitivity is technically challenging. The problem of low CTC numbers is more accentuated in brain tumors due to the blood–brain barrier, host immunity suppressing tumor migration, and survival outside the neuroaxis [7,169].

Nowadays, the study of CTCs has been performed in different types of tumors including lung [170], breast [171], prostate [172], and colorectal cancers [173], among others. The clinical utility of CTCs has been deeply investigated [171].

Globally, there are several approaches used to quantify CTCs. This generally starts with the CTC enrichment step, followed by either the positive selection of CTCs and/or the negative selection of leukocytes. Negative-enrichment techniques are essentially based on physical properties such as size, electric charge, or density. The positive selection of CTCs can be achieved by the use of immunological identification, which can detect specific tumor markers expressed on the surface of CTCs [174].

The Cell-Search^®^ system (Veridex) is the most common positive selection approach. It is currently the only clinically validated, FDA-cleared system for the identification, isolation, and enumeration of circulating tumor cells (CTCs) from a simple blood test. It is based on the use of antibodies against the transmembrane glycoprotein epithelial cell adhesion molecule (EpCAM), which is highly expressed in proliferating carcinomas [175]. To evaluate the cell viability of CTCs after separation, the use of the nuclear fluorescent dye 4,6-diamidino-2-phenylindole (DAPI) is essential. An antibody against cytokeratin or the intermediate filament of epithelial cells is also used (positive markers). In addition, a negative marker such as anti-CD45 is required. Eventually, the CTCs will be identified as DAPI+/CK+/CD45- cells. Cell-Search^®^ system is used in the monitorization of metastatic prostate [176], breast [177], and colorectal cancer [178]. However, non-epithelial cancers do not express epithelial biomarkers. Glioblastoma cells do not express EpCAM and, therefore, are not detectable using this Cell-Search^®^ approach. The isolation of CTCs from non-epithelial tumors is dependent on less specific microfluidic techniques that isolate cells based on physical properties and non-conventional methods [179] (Table 1).

Although the metastatic spread of glioma is extremely rare, several studies have reported the detection of circulating tumor cells with glioma characteristics.

Sullivan et al. reported the presence of rare circulating brain tumor cells within the peripheral blood in patients with glioblastoma. They proceeded to a “negative-depletion” through a CTC-iChip using a cocktail of antibodies specific to GBM markers (CD14, CD16, and CD45). CTCs were identified in at least one blood specimen from 13/33 GBM patients (39%) at different times in the treatment schedule. The isolated CTCs demonstrated an enrichment for mesenchymal over the neural differentiation markers compared with primary GBMs [190].

Mac Arthur et al. developed a strategy to detect CTCs based on telomerase activity. This technique offers high sensitivity, as more than 90% of solid tumors express elevated levels of telomerase, and high specificity, as telomerase is not found to be expressed in normal cells. The authors found that CTCs significantly reduced following radiotherapy. They suggest that this assay might assist the interpretation of treatment response in patients receiving radiotherapy and monitor disease recurrence [191].

Müller et al. identified CTCs using an anti-glial fibrillary acidic protein (GFAP) antibody. The authors identified CTCs in the blood of 30 out of 147 patients. The tumor origin of these cells was confirmed by the identification of shared mutations in the MDS1 and EVI1 complex locus, myosin 11, and neurofibromin 1, as well as the platelet-derived growth factor receptor Alpha and the amplification of the endothelial growth factor receptor [192].

Gao et al. developed an advanced integrated cellular and molecular approach of Immunostaining-FISH to detect CTCs in the peripheral blood (PB) of patients with seven different pathologic entities of primary gliomas (grade II-IV). The CTCs presented a strong polyploidy of chromosome 8 (≥5 copies) and a negative expression of both GFAP and CD45. The authors identified CTCs in the blood of 24 out of 31 (77%) patients with glioma in all seven subtypes. The study revealed the importance of CTCs in monitoring the treatment response and differentiating radionecrosis from the recurrence of glioma. CTCs can be used to complement radiographic imaging [193].

Krol et al. captured CTCs with a size-based antigen-agnostic approach, while red and white blood cells flowed through the device. The assessment of CTC presence and composition in 13 GBM patients was made during an open-label phase 1/2a study with the microtubule inhibitor BAL101553 [194].

In a more recent study, the authors examined CTCs collected from the peripheral blood of patients undergoing GBM resection. Cytomorphology was used for the size-based enrichment CTCs and their origin was confirmed based on mutational analysis. Next-generation sequencing (NGS) was performed for the identification of CTCs. The GeneReader™ sequencing platform allows the targeted sequencing of a 12-gene panel. The NGS approach in this study presents a major advantage of simultaneously identifying several markers relevant for GBM diagnostics, allowing more accurate diagnostics and the potential administration of innovative targeted therapies [195].

### 3.2. Circulating Cell-Free DNA

Mandel et al. revealed in 1948, for the first time, that tumors release nucleic acids into body fluids. Later, circulating DNA was shown to carry the same molecular information as tissue biopsies, opening up the possibility of using circulating DNA as a potential biomarker for diagnosing and monitoring cancers [196].

Circulating cell-free DNA (ccfDNA) are DNA fragments that circulate in body fluids at low concentrations (1–10 ng/mL). In normal conditions, ccfDNA is released during processes of cellular apoptosis or inflammation. In cancer patients, DNA fragments released by necrotic and/or apoptotic cells are called circulating tumor DNA (ctDNA) [197]. The total amount of ctDNA represents less than 0.1% to 5% of total ccfDNA and correlates with tumor type, grade, and burden. ctDNA exhibits somatic genetic alterations such as single nucleotide variants, chromosomal rearrangements, or gene copy number variations. However, due to the low concentration of ctDNA, the detection technology has to be highly sensitive and specific to distinguish ctDNA from normal leucocyte DNA. Conventional sequencing methods such as Sanger sequencing and pyrosequencing cannot always identify mutations in ctDNA, especially in cases of low-grade tumors. Furthermore, the mean half-life duration of ctDNA is short, varying from 1.5 to 2 h. All these facts make the detection of ctDNA extremely challenging [198]. Actually, two different approaches are used for ctDNA analysis. The first one is based on mutation-targeted analysis, and includes droplet digital PCR (ddPCR), BEAMing (beads, emulsion, amplification, and magnetics), the amplification refractory mutation system (ARMS), PCR, or PNA Clamp technologies [199]. The second approach is the use of next-generation sequencing (NGS). While ddPCR and BEAMing technologies allow the detection of low amounts of ctDNA (around 0.01%), the sensitivity is limited when using ARMS PCR or PNA Clamp techniques: it does not exceed 0.1%. On the other hand, NGS technology allows the exploration of a wide array of mutant DNA sequences present in a sample with high sensitivity and reproducibility. Furthermore, unlike previous techniques, NGS can detect novel and unknown genetic modifications [200].

Several studies have shown the possibility of the detection of tumor-associated mutations in ctDNA in patients with primary CNS tumors, including GBM. An initial study detected IDH1R132H substitution in the plasma samples of 80 glioma patients and 31 healthy controls. Fifteen out of twenty-five mutated tumors exhibited the mutation, and no mutation was detected in the fourteen patients with nonmutated tumors. The sensitivity increased in high-grade gliomas (WHO grades III and IV) and with enhancing tumor volume [201]. In 2014, Bettegowda et al. reported that ctDNA was detected in a limited subset (less than 10%) of patients with gliomas, significantly less than the other cancer types included in this study [202]. Piccioni et al. and Zill et al. identified ctDNA mutations in 55% and 51% of blood samples collected from GBM patients, respectively, using multigene sequencing platforms [203,204]. However, due to the low amount of ctDNA in the plasma of patients with primary brain tumors, CSF became an ideal alternative for highly sensitive ctDNA detection. Wang et al. analyzed CSF-tDNA in 35 primary CNS malignancies, using targeted or genome-wide sequencing. They identified detectable levels of CSF-tDNA in 74% of cases.

Martínez-Ricarte et al. performed the genomic analysis of IDH1, IDH2, *TP53*, TERT, *ATRX*, H3F3A, and HIST1H3B gene mutations in the CSF ctDNA of patients with diffuse gliomas [205]. They concluded that the analysis of these seven genes in CSF ctDNA facilitated the diagnosis of diffuse gliomas and supported surgical and clinical management. Indeed, the possibility of the detection of ctDNA in the blood and CSF samples allowed the tracking of specific alterations during treatment. It is from this perspective that Wang et al. (2015) tried to detect the methylation status of the MGMT promoters of 89 GBM patients using methylation-specific PCR. They found a higher sensitivity of detection when using CSF compared to serum samples (33.3% and 21.3%, respectively). Consequently, ctDNA provides a valuable option of pursuing and monitoring treatments.

Pan et al. performed a deep sequencing of glioma-associated genes on CSF-derived ctDNA from 57 patients with primary CNS tumors and compared the results with blood and tumor DNA. The study indicated that mutation detection using plasma ctDNA is less sensitive than sequencing the CSF ctDNA, and concluded that it is an alternative approach to stereotactic biopsy for detecting tumor-specific alterations in brainstem tumors [206].

Millet et al. evaluated the representation of the CSF genome from 85 glioma patients. ctDNA was detected in the CSF of 49.4% patients [207]. Despite the fact that the alterations detected in the CSF ctDNA of the patients closely resembled those in the tumor biopsies, the authors noted an important evolution in the growth factor receptor signaling pathways, leading to the possibility of monitoring the evolution of the tumor genome and using genotype-directed therapies through a minimally invasive technique.

Bagley et al. performed a prospective cohort study of 42 GBM patients [208]. They confirmed, once again, on a prospective cohort study of 20 GBM patients, that plasma cfDNA can be an effective noninvasive biomarker of tumor burden and prognosis. They also suggested cfDNA as a substrate for molecular profiling that can complement tissue sequencing.

A more recent study was conducted by Sheng et al. on 10 patients with glioma grade III or IV [209]. They proved the feasibility of characterizing the genomic landscape of these tumors using ctDNA extracted from tumor in situ fluid (fluid at the local surgical cavity) by analyzing the concordance between TISF and the tumor tissue results.

### 3.3. Cell-Free RNA

Cell-free RNAs are another group of molecules released from cells and tissues into body fluids. Their origin could be from passive secretion from necrotic or apoptotic cells, or active secretion through membrane-bound vesicles or a vesicle-free RNA-binding protein dependent pathway. There are different kinds of cell-free RNAs (cfRNAs). Globally, we distinguish coding RNAs (messenger RNAs) and noncoding RNAs (ncRNAs). Several studies have shown that the quasi-majority of the transcriptome corresponds to ncRNAs, and less than 2% of the human genome is transcribed into protein-coding RNAs [210]. This fact, together with the high stability of cf-ncRNAs (due essentially to their presence inside vesicles or their association with others proteins), has greatly increased the interest of studying their potential role as diagnosing or prognosing cancer biomarkers [211].

There are two classes of ncRNAs based on transcript size: small ncRNAs that are shorter than 200 nucleotides, and long non-coding RNAs (lncRNAs) that are longer than 200 nucleotides. The small ncRNAs include microRNAs (miRNA), short interfering RNAs (siRNAs), piwi-interacting RNAs (piRNAs), and small nucleolar RNAs (snoRNAs). The lncRNAs include intronic lncRNAs, intergenic lncRNAs (lincRNAs), enhancer lncRNAs (elncRNAs), bidirectional lncRNAs, sense-overlapping lncRNAs, and antisense lncRNAs [212,213,214].

MicroRNAs are the most studied ncRNA molecules. Their length is between 18 and 25 nucleotides. They play an important role in post-transcriptional gene regulation due to the fact that a single miRNA might affect different mRNAs and, in the same way, one mRNA could contain target sites for different miRNAs. Their role in proliferation, development and apoptosis is also well established. Many cancers are associated with altered miRNA expression. Eventually, cf-miRNAs have come to be considered useful cancer biomarkers thanks to their relatively high diagnostic value, as proven by several studies [215,216]. A systematic review of the expression profiles and function of miRNA identified 253 upregulated and 95 downregulated profiles in GBM patients. Both oncogenic and tumor-suppressive miRNAs were found to affect target genes involved in different processes such as cell migration, invasiveness, and angiogenesis [217]. Other studies have tried to functionally describe and associate some miRNAs with the survival or disease progression of GBM patients [218,219]. Since glioblastoma shows mostly resistance to radiotherapy treatment, numerous miRNAs in connection with pathogenesis and the radio-responsive state have been studied to identify their role and their potential as radiosensitizing agents in GBM [220].

Mir-21 is the most investigated miRNA in cancer and its overexpression has been consistently reported in the tissue and the plasma of GBM patients [221,222,223]. In fact, several studies have associated miR-21 with tumor grading and lower overall survival. In 2019, Wang J et al. conducted a meta-analysis of 47 studies including 2262 glioma patients and 1986 controls. They reported that cell-free microRNAs were relatively efficient in diagnosing gliomas, with a sensitivity of 83% and a specificity of 87%. Like previous studies, miR-21 was the best biomarker, followed by miR-125 and miR-222 [224]. Zhi et al. found that the upregulation in the serum miR-20a-5p, miR-106a-5p, and miR-181b-5p was significantly associated with high-grade astrocytoma, and the high expression of serum miR-19a-3p, miR-106a-5p, and miR-181b-5p was associated with poor patient survival [225]. Zhao et al. performed global miRNA profiling in serum samples of 106 primary glioblastomas. They also identified that elevated levels of serum miR-106a-5p and miR-20a-5p were associated with poor overall survival and disease-free survival [226].

Alongside the miRNA class, the lncRNA class is presented as a potential cancer biomarker. Indeed, lncRNAs are found to be overexpressed in gliomas and their signature has been associated with overall survival in patients with glioblastoma [227]. Gal Mazor et al. found that TP73-AS1 is a clinically relevant lncRNA in GBM. They observed the significant overexpression of TP73-AS1 in primary GBM samples. They demonstrated that TP73-AS1 constitutes a strong prognostic biomarker since lncRNA promotes tumor aggressiveness and TMZ resistance in GBM cancer stem cells [228].

Numerous previous studies have evaluated the diagnosis and/or prognosis value of the long noncoding RNA HOX transcript antisense intergenic RNA (HOTAIR). Shen et al. observed high levels of HOTIAR and low levels of GAS5 in the serum of GBM patients. These levels were associated with a reduced probability of 2-year survival [229]. Meanwhile, Tan et al. revealed higher levels of HOTAIR expression in serum isolated from GBM patients compared to controls. These levels were positively correlated in both tumor and serum samples. They demonstrated that HOTAIR can be used as a peripheral biomarker for detecting GBM [230]. Zhihai Yuan et al. also reported that LncRNA HOTAIR was significantly upregulated in TMZ-resistant glioblastoma cells. They indicated that exosomal lncRNA HOTAIR induced TMZ resistance and modulated TMZ resistance through the miR-519a-3p/RRM1 axis. Furthermore, serum exosomal HOTAIR had a good diagnostic value [231]. These results were confirmed in other studies [231].

Globally, most of cfRNA assays are still exploratory, and the need for clinical studies with standardized protocols is essential before integrating these molecules in the clinical management of glioblastoma.

### 3.4. Extracellular Vesicles

Extracellular vesicles (EVs) are a group of small membrane-enclosed spheres released by cells. EVs can be classified into two categories depending on their biogenesis: exosomes produced initially in the cytoplasm with an intact endosomal membrane before fusing with the plasma membrane and which are released from the cell via exocytosis; and microvesicles that shed directly from the extracellular membrane via budding. EVs can also be classified depending on their size, density, and cargo [232,233]. EVs contribute to intercellular communication by carrying cell components including proteins, membrane lipids, cell metabolites, and nucleic acids. Thus, the production of EVs is ubiquinone, and tumor cells are very active in secreting EVs, especially during cancer progression, by mediating the transportation of various factors contributing to the control and deregulation of proliferation, drug resistance, migration, angiogenesis induction, and invasion [234]. Unlike ctDNA, EVs can arise from viable cancer cells and hold the capacity to protect their contents from enzyme degradation, enabling further studies [235].

Many papers have shown that glioma—and particularly glioblastoma—cells are capable of producing EVs with molecular composition to characterize tumor cells. Moreover, EVs can cross the blood–brain barrier, both in physiological and pathological conditions, making them detectable in the bloodstream and CSF [236].

Skog et al. have shown that EVs can be isolated from the serum of glioma patients, identifying EGFRvIII mRNA in 7 out of 25 GBM patients. Furthermore, the authors found that no EGFRvIII was detectable after 2 weeks of resection, which suggests a direct relationship with tumor burden [237]. Other genes and gene products with smaller alterations, such as mutant *IDH1* protein, were detected in glioma patient sera and cerebrospinal fluid extracellular vesicles [66,238].

Akers et al. distinguished miR-21 [239] and a microRNA signatures [239] in the extracellular vesicles of the cerebrospinal fluid of patients with GBM.

More importantly, extracellular vesicles might assist in the clinical diagnosis and prognosis of patients with GBM [9]. Osti D et al. noticed reduced levels of EVs after resection and higher levels at recurrence. The protein cargo of EVs could provide indications about the tumor, therapy response, and monitoring [240]. Numerous studies have reported that treatment with temozolomide may affect EV release and confer drug resistance. As an example, EVs collected from TMZ-resistant patients showed increased expression levels of MGMT [241]. Therefore, analyzing the molecular components of EVs could help monitor temozolomide treatment [242,243].

Several commercial kits are used to purify extracellular vesicles [168]. Van Deun et al. provided a comparative evaluation of four exosome isolation protocols and showed that OptiPrep^®^ density gradient centrifugation outperformed ultracentrifugation, ExoQuick^®^, and Total Exosome Isolation^®^ precipitation in terms of purity [244]. Saenz-Cuesta et al. demonstrated that the highest EV concentrations were obtained using the ExoQuick^®^ protocol by comparing five different protocols [245]. Generally, there is no standard method to isolate exomes or to discriminate tumor and non-tumor exosomes, which is limiting their use in clinical practice.

### 3.5. Circulating Proteins

Circulating proteins can either be detected in blood or the CSF of patients with brain tumors. Protein isolation is the most commonly studied technique, mainly due to its relatively low cost. Nevertheless, since no glioma-specific proteins have been identified, studies globally rely on detecting altered levels of some circulating proteins that are derived from tumor cells [197].

GFAP is one of the most detected proteins highly expressed in glioblastoma patients. Indeed, serum GFAP levels are correlated with tumor volume and histopathological tumor characteristics [246]. Pérez-Larraya et al. proposed a combined profile analysis of IGFBP-2, GFAP, and YKL-40 plasma levels, and suggested it as an additional diagnostic and prognostic tool, especially for patients with inoperable brain lesions [247]. Furthermore, the measurement of the expression of the seven markers involved in the angiogenesis and inflammation pathways of patients with glioblastoma allowed them to establish statistically significant associations between the presence of low levels of IL-8 and the development of coagulation necrosis, high levels of VEGF, the development of ischemic necrosis and high levels of IL-8, and the development of endothelial hyperplasia [248].

Moreover, since YKL-40 is one of the most highly expressed genes in glioblastoma, Qin et al. conducted a meta-analysis and found a strong association between high YKL-40 expression and worse overall survival in glioblastoma patients, making YKL-40 a potential good biomarker of prognosis [249].

A more recent study reported alterations in other aspects of the proteome. Quantitative comparisons of the plasma proteomes of GBM patients and healthy controls were performed using SWATH (Sequential Windowed Acquisition of All Theoretical Fragment Ion) mass spectrometry analysis. As a result, eight biomarker candidates for GBM were identified. Among them, leucine-rich alpha-2-glycoprotein (LRG1), complement component C9 (C9), and C-reactive protein (CRP) showed significant positive correlations with tumor size [250].

In 2020, Naryzhny et al. tried to define the common exosomal proteins presented in preparations from different cell lines and determine the potential glioblastoma biomarkers in exosomes. Using the proteomic analysis of the exosomes, they generated a list of 133 proteins common for all of the samples analyzed. A correlation between some of these proteins overexpressed in glial cells and their presence in exosomes has been established, confirming the existence of many potential glioblastoma protein biomarkers in exosomes [251].

## 4. Conclusions and Future Insights

Despite major advances in understanding the pathogenesis of glioblastoma, patients are still facing poor overall survival and limited treatment options. The current diagnosis of glioblastoma is mainly based on the histo-molecular study of tissue biopsies combined with imaging. Several genetic techniques, including next-generation sequencing, have been used to explain the signaling pathways and to determine the biomarkers characterizing the diagnosis and prognosis status of these tumors. For instance, IDH, *TP53*, PTEN, *ATRX*, TERT mutations, and EGFR amplification are some influential molecular biomarkers that have shown a clinical impact regardless of the lack of improvement of the patients’ overall survival. Therefore, it is crucial to integrate molecular analysis into the clinical process.

This standard protocol has many limitations. Indeed, tissue biopsies are generally invasive and cannot easily be repeated. Conventional MRI may guide the surgeon, but sometimes presents results that are difficult to interpret. Over the past decade, liquid biopsy research has led to the development of new cancer management procedures for more personalized medicine in oncology. In fact, liquid biopsy gives the possibility of taking repetitive and non-invasive samples, which allows real-time monitoring of the patient during treatment. A liquid biopsy can sometimes reveal information about the tumor even before clinical progression. Nevertheless, the histology of the tumor and the characteristics of its microenvironment are mainly provided by tissue biopsy. Therefore, liquid biopsy should be considered as a complementary tool to current procedures, allowing better follow-up and optimal management of the patient. Serum, CSF, and other body fluids carry biomarkers such as CTCs, nucleic-acids, extracellular vesicles, and circulating proteins that are linked to diagnosis and/or prognosis. Moreover, some of these particles might be used to detect therapeutic resistance or identify tumor recurrence. However, to date, no circulating biomarkers for managing GBM have been clinically validated. As each biomarker has advantages and disadvantages, a combination of biomarkers could be valuable to obtain diagnostic and prognostic information in a non-invasive method. Nonetheless, further studies with larger cohorts are needed in order to increase specificity and sensitivity, and to promote future clinical applications.

## Figures and Tables

**Table 1:** Some commercial technologies for the detection of circulating tumor cells.

Methods	Commercial Kit	Key Features	Tumor Type	Clinical Utility	Limits	Ref
Immune-Based Detection						
Magnetic nanoparticles	Cell-Search^®^	Positive (EpCAM) and negative (CD45) selection followed by Immunofluorescent staining of CKs and DAPI; Only FDA approved; Most clinically validated platform for the detection of CTCs.	Metastatic Breast; Colorectal; Prostate	Prognosis; Treatment	Reliance on specifically expressed protein markers; expensive; low cell viability	[176,179]
	AdnaTest^®^	Antibodies against epithelial and tumor-associated antigens conjugated to magnetic beads for the labeling of tumor cells in whole blood; Positive selection followed by RT-PCR analyses; Analyzes clinical relevance by testing the gene expression of specific tumor markers.	Breast; Prostate; Ovarian; Colon	Prognosis; Treatment		[180,181]
Microfluid device	GEM chip	Mixing chip structure for enhanced capture of CTC on antibody-coated surfaces.	Pancreatic	Diagnosis; Treatment	Limited sample volume; Slow flow rate.	[182]
Dual Modality	Ephesia (CTC-chip)	Magnetic beads coated with EpCAM antibodies self-assemble into a periodic array under a high magnetic field.	Lung; Breast; Colorectal; Prostate	Diagnosis; Prognosis	Moderate sensitivity; Expensive technology	[183]
**Biophysical properties**						
Size	ISET^®^ (Rarecells Diadnostics)	Filtration system to eliminate white blood cells (WBC) followed by the enrichment of CTCs, cytomorphology analyses, and immunostaining characterization	Breast; Melanoma; Lung; Hepatocellular carcinoma	Prognosis; Treatment	Difficulty in the detachment of CTCs from the filter; Different sizes of CTCs	[184]
Electric charge	ApoStream^®^	Isolation of CTCs based on cell differences in dielectric properties (polarizability) using a process called dielectrophoresis (DEP) field-flow assist in a microfluid chamber	Breast	N.A	Cell electrical properties can be affected during the procedure; Large number of parameters to control simultaneously	[185,186]
Density	OncoQuick^®^	Isolation of CTCs using porous membrane filtration followed by density gradient centrifugation	Gastrointestinal; Breast	Prognosis	Loss of large CTCs and cell aggregates	[187]
Imaging	FASTcell (SRI)	An array of optical fibers provides a larger field-of-view than traditional optical systems; Reduces time for imaging using a laser light source and a sensitive photomultiplier detector	Breast; Lung	Prognosis; Treatment	Difficult sample processing; Loss of cells under investigation	[188]
Inertial focusing	Vortex	Selective capture of CTCs using micro vortices and inertial focusing	Breast; Lung	Diagnosis; Prognosis; Treatment	Complicated principle; Morphological deformation of captured cells	[189]

## Data Availability

Not applicable.
